# Bio-inspired neural networks with central pattern generators for learning multi-skill locomotion

**DOI:** 10.1038/s41598-025-94408-0

**Published:** 2025-03-24

**Authors:** Chuanyu Yang, Can Pu, Yuan Zou, Tianqi Wei, Cong Wang, Zhibin Li

**Affiliations:** 1 National Elite Institute of Engineering, Chongqing University, Chongqing, 401135 China; 2 Shenzhen Amigaga Technology Co Ltd., Shenzhen, 518000 China; 3 School of Artificial Intelligence, Sun Yat-sen University, Zhuhai, 519082 China; 4 Shenyang Institute of Automation, Chinese Academy of Sciences, Shenyang, 110003 China; 5 Department of Computer Science, University College London, London, WC1E 6BT UK

**Keywords:** Machine learning, Learning algorithms, Central pattern generators, Engineering, Computer science

## Abstract

Biological neural circuits, central pattern generators (CPGs), located at the spinal cord are the underlying mechanisms that play a crucial role in generating rhythmic locomotion patterns. In this paper, we propose a novel approach that leverages the inherent rhythmicity of CPGs to enhance the locomotion capabilities of quadruped robots. Our proposed network architecture incorporates CPGs for rhythmic pattern generation and a multi-layer perceptron (MLP) network for fusing multi-dimensional sensory feedback. In particular, we also proposed a method to reformulate CPGs into a fully-differentiable, stateless network, allowing CPGs and MLP to be jointly trained using gradient-based learning. The effectiveness and performance of our approach are demonstrated through extensive experiments. Our learned locomotion policies exhibit agile and dynamic locomotion behaviors which are capable of traversing over uneven terrain blindly and resisting external perturbations. Furthermore, results demonstrated the remarkable multi-skill capability within a single unified policy network, including fall recovery and various quadrupedal gaits. Our study highlights the advantages of integrating bio-inspired neural networks which are capable of achieving intrinsic rhythmicity and fusing sensory feedback for generating smooth, versatile, and robust locomotion behaviors, including both rhythmic and non-rhythmic locomotion skills.

## Introduction

Legged robots have been increasingly deployed for applications in complex, diverse, and unstructured environments, requiring robust and adaptive locomotion strategies. Achieving such capabilities is challenging due to the need to coordinate multiple degrees of freedom and generate effective motion patterns to adapt to environmental uncertainties.

In recent years, there has been significant progress in using deep reinforcement learning (DRL) to train artificial neural networks for legged locomotion^1,2^. A common type of artificial neural network is the Multi-Layer Perceptron (MLP), also referred to as a Fully Connected Neural Network. An MLP consists of an input layer, one or more hidden layers, and an output layer. Each layer is composed of neurons that are connected to the neurons in the preceding and subsequent layers. Each connection between two neurons is associated with a weight. Each neurons in the hidden and output layers applies an activation function to its incoming weighted sum^3^. Multi-expert neural networks can be designed to perform various locomotion tasks^1^. Robust control policy has also been trained to successfully traverse over extreme terrain in the wild^2^. The neural networks from both works consists of MLPs and rely on an external periodic phase input to produce rhythmic motor patterns. Yang et al. utilized a global phase input consisting of a sine and cosine pair that represents a 2D-unit cycle, while Lee et al. provided local phase inputs for each leg represented by a single value that linearly increments from 0 to $2\pi$ and resets^1,2^. A dedicated component has to be implemented to provide the external phase, whether it is achieved through manual design or through the learning capabilities of a neural network. However, incorporating an explicit periodic phase input is not trivial, as such an external phase requires either manual design or additional learning components, both of which complicate the design and training process.

**Fig. 1:**
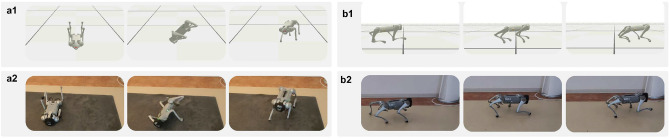
Depiction of the Go1 robot performing (**a1**) fall recovery in simulation (**b1**) locomotion in simulation (**a2**) fall recovery in real world (**b2**) locomotion in real world.

To address the limitation of providing external periodic phase input, there have been attempts in designing network structures capable of self-generating phases or removing phases altogether^4,5^. One approach is to add an additional phase increment output to the policy. The phase increment is added to the current phase and is used as the phase for the next timestep in a bootstrap manner^2,6,7^. Another approach is to manually design a trajectory generator that is capable of modulating phase according to sensory feedback and learn a policy to generate residual corrections^8^.

### CPG-based controllers in robotics

While various DRL approaches require external phase input, an increasing interest is shifting towards self-generating phase internally to avoid the need of phase inputs, for which Central Pattern Generator (CPG) comes into play.

Animals, for example, are able to adapt their locomotion gait patterns according to the travelling velocity and ground conditions. Efforts have been made on discovering the underlying mechanism of animal locomotion, and there are evidence that legged locomotion is rhythmic in nature as findings have revealed the existence of special neurons called central pattern generators (CPG) in the animal spinal cord. CPGs are a series of coupled oscillatory neurons capable of producing rhythmic signals internally without the external sensory input. The rhythmic signals produced by CPGs are necessary for activities that involve periodic movements, such as breathing, walking, running, swimming, flying, etc.^9,10^.

CPGs have gained interest within the robotics field due to their oscillatory and rhythmic nature. To study the rhythmic mechanism of biological CPGs, researchers have proposed different CPG models with varying levels of complexity, from detailed biophysical models, and connectionist models, to abstract models based on mathematical oscillators^10,11^.

Researchers have attempted to leverage the natural rhythmic behaviors of CPGs by incorporating CPGs into the controller to achieve animal-like locomotion for all kinds of robots^12–14^. Various abstract models of mathematical oscillators such as SO(2) oscillator, Matsuoka oscillator, and Hopf oscillator have been employed within the locomotion controller for different robots^15–17^. CPG controllers are usually integrated with sensory feedback to react to external environmental stimuli. The feedback loop can either be manually designed with simple rules inspired by biological reflex^18,19^, or be a trained neural network for more complex reflex behaviors^17^. Degallier et al. and Ajallooeian et al. have utilized ground contact as sensory information, while Gay et al. have utilized a more diverse set of sensory information that includes readings from gyroscope and optical flow.

### Learning CPG-based controllers with DRL

DRL has proven to be effective in learning robust and complex locomotion skills for legged robots in simulation and real world. Incorporating CPG-based controllers within the DRL paradigm offers a promising solution that enables robots to learn from the interaction with the environment^20–23^. However, due to the intrinsic recurrent nature of CPG networks, back-propagation through time (BPTT) is required to obtain the parameters of CPGs while trained with gradient-based learning, which is computationally more expensive^22,24^.

One approach of dealing with the recurrent nature of CPGs is to treat the CPG controller as part of the environment dynamics. Such an approach is referred to as CPG-Actor-Critic, where the CPG controller, the robot, and the environment are treated as a single dynamic system called CPG-coupled system. The action space of the policy is the CPG parameters. The policy controls the robot by adjusting the CPG parameters of the CPG-coupled system^20–23^.

Another approach to dealing with the recurrent nature of CPGs is to optimize the parameters of CPGs and MLP network separately. The parameters of the neural network can be optimized with DRL, while the parameters of CPG are optimized using non-gradient-based approaches such as evolutionary strategy (ES)^25^, genetic algorithm (GA)^26^, or a biologically plausible learning rule^27,28^. Wang et al.^26^ proposed a hierarchical control structure with a high-level neural network and a low-level CPG controller. The high-level network generates latent variables that are fed into the CPGs to regulate their behaviors. The CPG controller is optimized using GA, while the neural network is trained using DRL. Shi et al.^25^ used ES to train a CPG-based foot trajectory generator. A separate neural network is trained using DRL to generate residual joint angles that correct the generated foot trajectory.

Both of these approaches require separate design and training of the CPG and MLP components, leading to additional complexity. This separation creates a significant burden in terms of design effort and training time.

### Multi-skill locomotion and fall recovery

In the real world, there exist extreme situations and uncertainties that cause the robot to inevitably fall over. The ability to recover from falls and presume normal locomotion is crucial. Lee et al.^2^ trained a locomotion policy and fall recovery policy separately, and utilized a high-level network to switch between different policies. Ji et al.^29^ combined a ball dribbling policy and fall recovery policy together with a fall detector. The fall detector switches between two policies. Smith et al.^30^ designed three control policies, each responsible for forward locomotion, backward locomotion, and fall recovery. Yang et al.^1^ achieved multi-skill locomotion via a multi-expert network structure, where locomotion and fall recovery maneuvers are controlled by different expert policies.

While these approaches are effective, they typically require the design and training of separate dedicated policies for locomotion and fall recovery. This approach can be labor intensive, as it involves additional manual efforts to ensure that each policy works optimally in different contexts.

### Contribution

This work aims to utilize the natural rhythmicity of CPGs to generate agile and dynamic locomotion behaviors for quadruped robots. While CPGs by themselves are capable of generating open-loop rhythmic signals, sensory feedback has to be integrated to enable the CPGs to react to the external environment. We propose a framework that combines MLP networks and CPGs, where the CPGs are responsible for generating rhythmic patterns, and the MLP receives sensory information and provides feedback to modulate the CPGs’ behavior.

The contributions of this paper are:

- A novel network architecture that combines CPGs and MLP network to generate rhythmic motions intrinsically. We name the network architecture MLP-CPG.
- A new mathematical formulation that reformulates CPGs into fully differentiable networks. The CPG parameters and MLP parameters within the MLP-CPG network architecture can be jointly optimized with DRL.
- Multi-skill motor capabilities through one single unified policy network, achieving both rhythmic locomotion and non-rhythmic fall recovery, as well as seamless transitions between these distinct motor behaviors.
- Demonstration of hardware feasibility of the proposed framework by successfully real robot performance. The robustness of the policy is validated through rigorous experimentation across diverse real-world terrain scenarios.

Compared to previous methods that depend on external phase inputs for rhythmic locomotion, our MLP-CPG framework eliminates this dependency by leveraging the natural rhythmicity of CPGs. Our proposed MLP-CPG framework reformulates CPGs by exposing the internal states as inputs and outputs of the network, essentially turning CPGs into a fully-differentiable stateless network. The parameters of the MLP and CPGs are optimized in conjunction via DRL in a coherent manner. Additionally, traditional approaches often employ separate policies for locomotion and fall recovery, requiring additional design of switching mechanisms. In contrast, our proposed MLP-CPG framework achieves versatile multi-skill motor capabilities of rhythmic locomotion and non-rhythmic fall recovery within a single unified network policy, enabling seamless transitions and reducing design complexity (Fig. 1).

The trained MLP-CPG policy demonstrates robustness while navigating diverse environmental uncertainties in simulation, and has been effectively validated on real robot hardware across a range of real-world terrain scenarios. The simulation and real world experimental results are available in the following videos: simulation results and real-world experiments.

## Results

### Network comparison

**Fig. 2:**
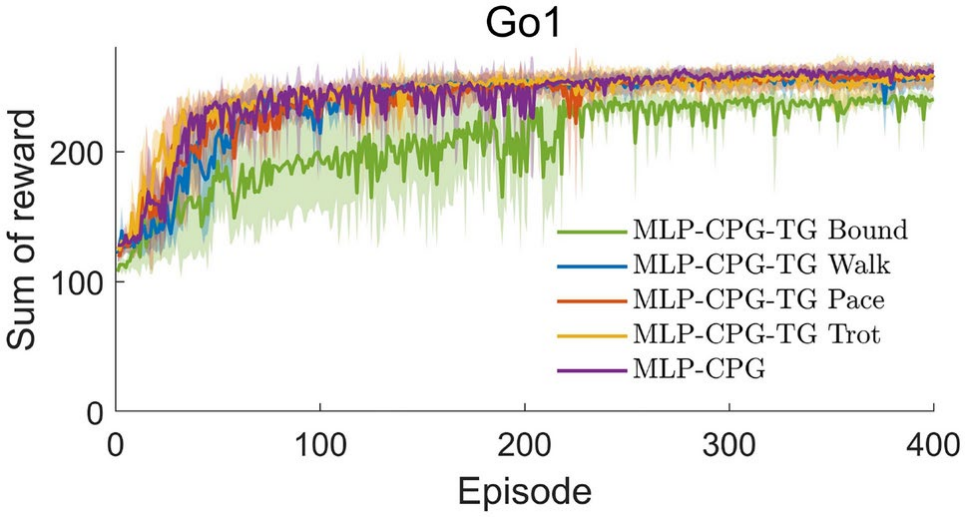
Learning curve of Go1. The results are averaged over 6 trials, each with a different random seed.

We conduct a comparison study where we handcrafted the parameters of CPG to match typical gait patterns. We refer to MLP-CPG network with handcrafted CPG parameters as MLP-CPG-TG (Trajectory Generator). We manually handcrafted the coupling parameters $\varvec{\varepsilon }$ and $\varvec{\phi }$ of three types of MLP-CPG-TG networks to generate typical gaits of pacing, trotting, and walking. The MLP-CPG-TG network resembles policy modulated trajectory generator (PMTG) in the sense that the handcrafted CPG serves as a trajectory generator, and the separate feedforward network serves to modulate the generated trajectory^8^. Both MLP-CPG-TG and PMTG require prior knowledge to be introduced in the form of a predefined trajectory generator, while our proposed MLP-CPG requires no prior human knowledge as the rhythmic CPG module learns the trajectory by itself. From the learning curve, we are able to see that the manually handcrafted MLP-CPG-TG with bounding gait has the lowest performance, while the other training configurations exhibit similar performance (Fig. 2).

**Table 1:** The mean and standard deviation of the absolute values of the normalized velocity tracking errors for five training setups each gathered from 6 training trials.

Network structure	$v_{x}$	$v_{y}$	$\omega _{yaw}$
MLP-CPG-TG-Bound	$0.098 \pm 0.069$	$0.339 \pm 0.190$	$0.584 \pm 1.885$
MLP-CPG-TG-Walk	$0.070 \pm 0.067$	$\varvec{0.294} \pm \varvec{0.187}$	$0.374 \pm 1.295$
MLP-CPG-TG-Pace	$0.060 \pm 0.060$	$0.543 \pm 0.390$	$0.700 \pm 4.674$
MLP-CPG-TG-Trot	$0.068 \pm 0.0.067$	$0.350 \pm 0.123$	$0.339 \pm 0.987$
MLP-CPG	$\varvec{0.055} \pm \varvec{0.061}$	$0.321 \pm 0.155$	$\varvec{0.298} \pm \varvec{0.779}$

The velocity tracking error is the difference between desired locomotion velocity and actual velocity of the robot base during simulation. The velocity tracking errors are normalized by their corresponding target velocities, therefore resulted in unitless quantities. Data are gathered by providing each policy with the same set of desired locomotion velocity. The lower the absolute tracking error value, the higher the performance. The terms $v_x$, $v_y$, $\omega _{yaw}$ are the forward linear velocity along the x axis, lateral linear velocity along the y axis, and yaw angular velocity of the robot base respectively. The velocities are represented in the robot base frame.

Significant values are in bold.

The velocity tracking performance is nearly identical among the five training setups. with MLP-CPG having slightly better tracking performance in $v_{x}$ and $\omega _{yaw}$, and MLP-CPG-TG-walk performing better in $v_{y}$. Among the four manually designed MLP-CPG-TG networks, the network with trotting gait has the best performance (Table 1). Overall, our MLP-CPG is able to achieve slightly higher performance than the hand-tuned MLP-CPG-TG with the benefit of less manual hand-tuning.

### Multi-skill behavior

Our approach achieves multi-skill motor behaviors of rhythmic locomotion and non-rhythmic fall recovery within a single policy, without the need for multiple separate policies or mixture-of-expert network structures.

**Fig. 3:**
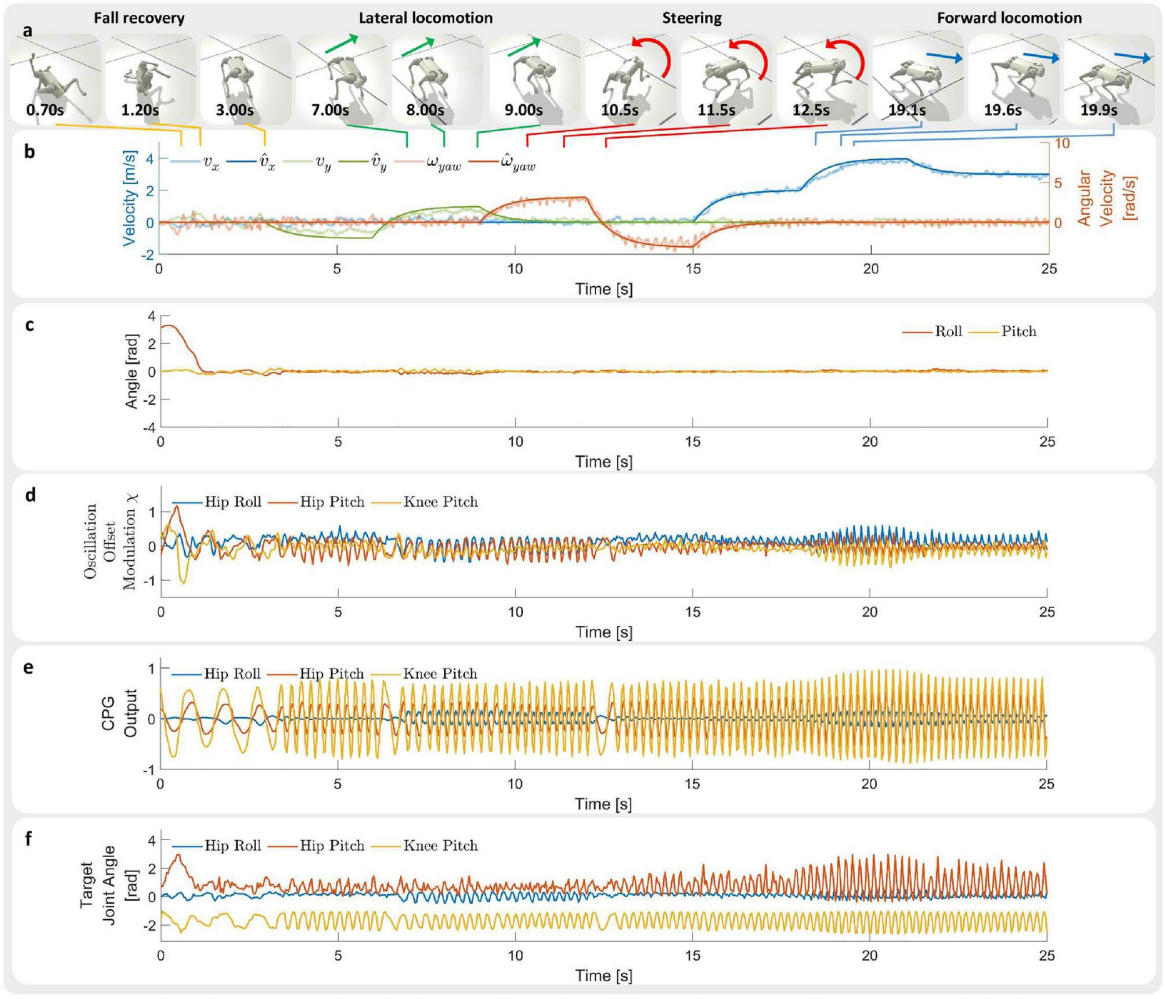
The robot is commanded to follow various velocity commands in simulation. Hip Roll represents the hip abduction/adduction joint, Hip Pitch represents the hip flexion/extension joint, and Knee Pitch represents the knee flexion/extension joint. (**a**) Snapshots of the robot performing fall recovery, lateral locomotion, steering, and forward locomotion in simulation. (**b**) Measured velocity and target velocity. (**c**) Roll and pitch angles of robot base. (**d**) CPG oscillation offset $\chi$ of front left leg joints. (**e**) CPG output signal $\psi$ of front left leg joints. (**f**) Target joint angles of front left leg joints. (see Supplementary Video S1 for the corresponding video. Video URL: https://youtu.be/JmZGazoHKDs).

The robot automatically determines whether it has fallen from proprioceptive inputs. The behavior is learned and is executed automatically without manual user command (Fig. 3). While the robot is performing fall recovery, we command the target velocity to be 0 to avoid unnecessary movement. The learned MLP-CPG policy is able to achieve fall recovery within 1.5*s* (Fig. 3c). The policy relies on modulating the oscillation offset $\chi$ to produce non-rhythmic movements necessary for fall recovery as can be seen from the large change in signal between time period $0{\text{s }}- 1.5{\text{s}}$ in Fig. 3d.

After a successful fall recovery, we command the robot to follow a series of different velocity commands (Fig. 3a). The simulated robot is able to track the target velocity (Fig. 3b). It can be seen that the policy adjusts the CPG amplitude in response to user target velocities. There is a significant increase in the amplitude of the Hip Pitch and Knee Pitch joints at high locomotion velocity of 4 m/s (Fig. 3e). The MLP network plays a large role in generating non-rhythmic movements as can be seen from the significantly larger offset values $\chi$ during fall recovery compared to locomotion (Fig. 3d and f). The network achieves both rhythmic locomotion and non-rhythmic fall recovery by modulating the CPG output and oscillation offset values $\chi$. During fall recovery, the amplitude and frequency of the CPG output decrease, accompanied by an increase in the oscillation offset, which results in the generation of non-rhythmic movements. In the opposite case during locomotion, the oscillation offset decreases, while the amplitude and frequency of the CPG output increases, which results in the generation of rhythmic movements.

### Performance benchmark

**Fig. 4:**
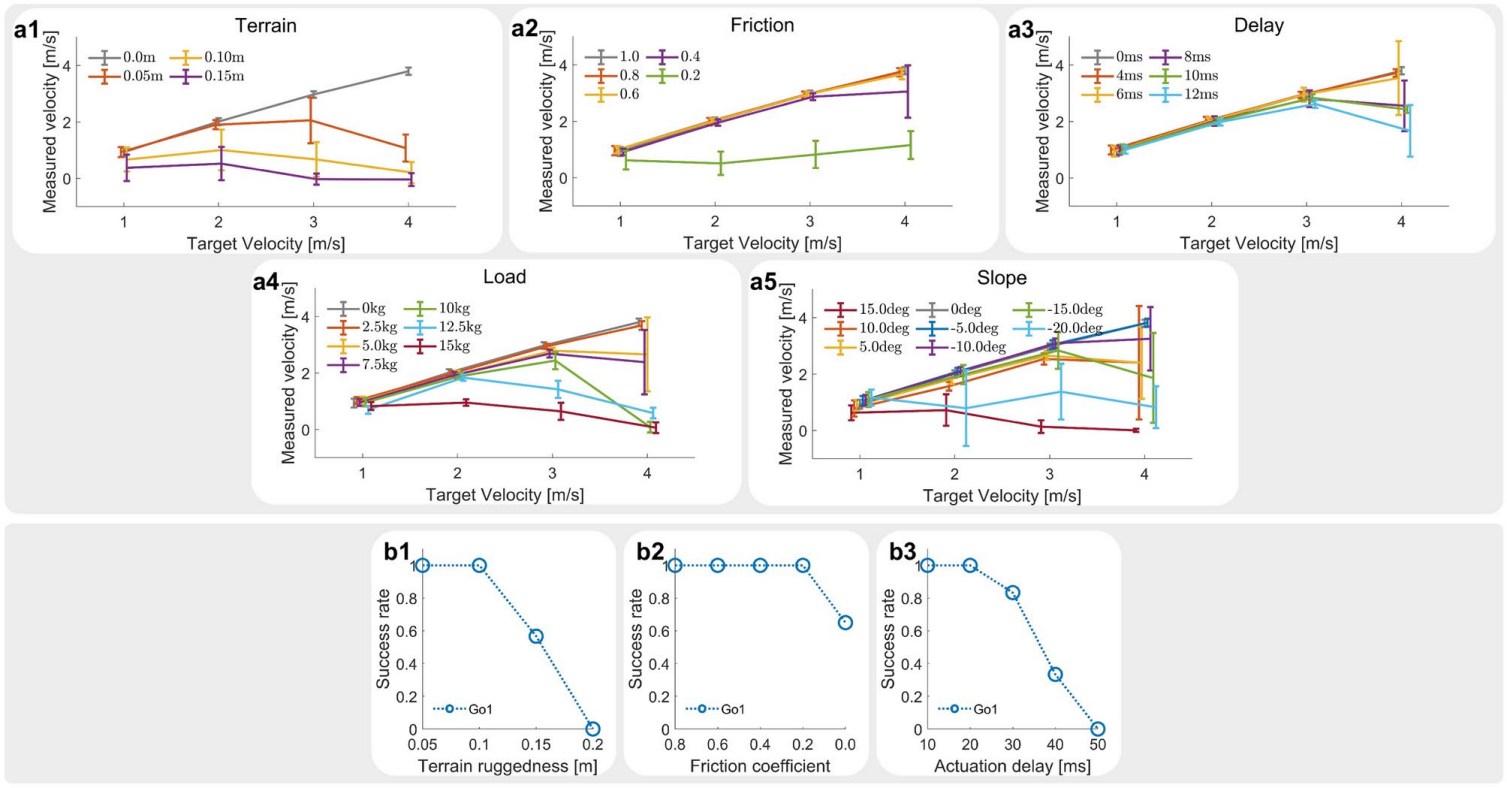
(**a**) Target velocity and measured velocity of the robot in different simulated terrain conditions. (**a1**) Uneven terrain. (**a2**) Low friction. (**a3**) Actuation delay. (**a4**) External load. (**a5**) Slope. (**b**) Success rate of fall recovery maneuvers of the robot in different simulated terrain conditions. (**b1**) Uneven terrain. (**b2**) Slippery surface. (**b3**) Actuation delay.

We evaluate the Go1 robots’ statistical performance on unseen terrain situations (Fig. 4). Fall recovery is evaluated using success rates. We confirm the success of the fall recovery motion when the robot satisfies the following three criteria: (i) Base height maintains higher than 0.3m, (ii) Base roll and pitch angle maintain within (-$\frac{\pi }{4}$,$\frac{\pi }{4}$), (iii) Only having feet in contact with the ground. For fall recovery, we design test scenarios of (1) Uneven terrain, (2) low friction, (3) actuation delay.

Locomotion performance is evaluated using the robot’s ability to track the target velocity. The robot is commanded to move in a straight line at a given speed. We observed that the higher the locomotion velocity, the more the Go1 robot is affected by external uncertainties. For locomotion, we design test scenarios of (1) Uneven terrain, (2) low friction, (3) actuation delay, (4) load carrying, (5) walking on slope.

**Fig. 5:**
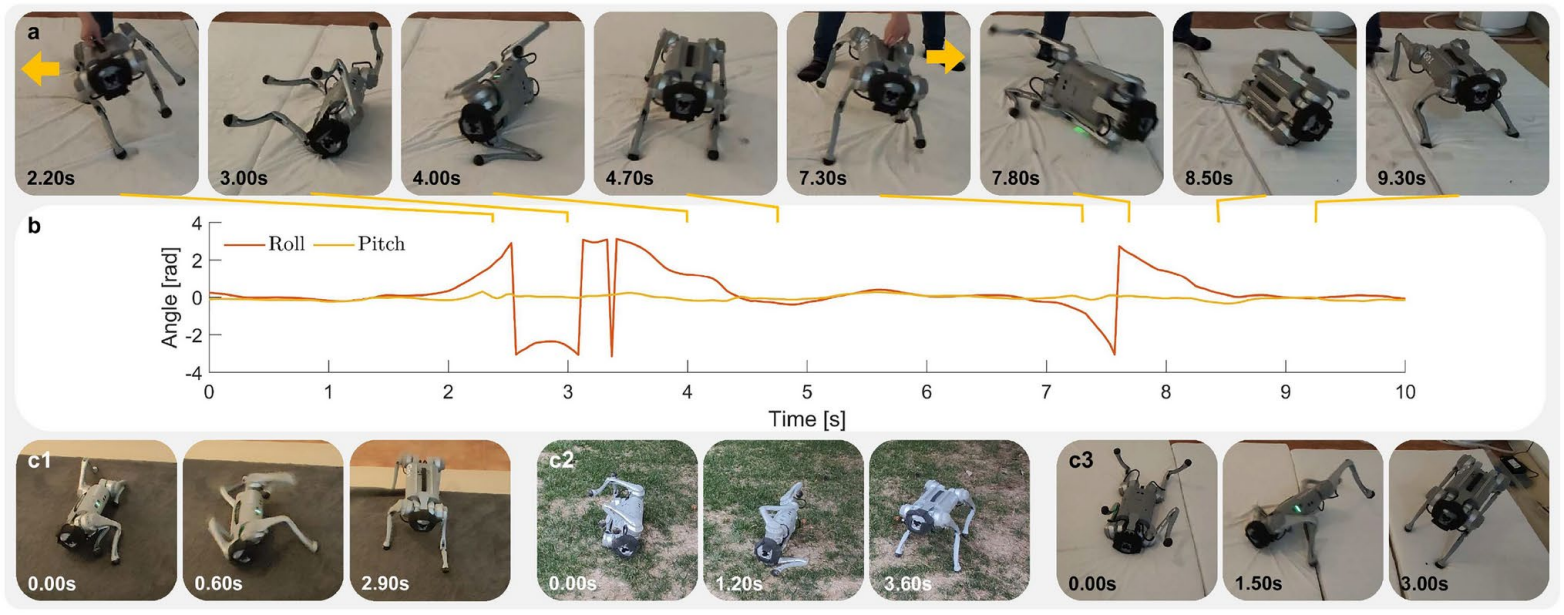
Robot recovering from fall in the real world. (**a**) Snapshots of the robot performing agile fall recovery motions after experiencing external disturbance. (**b**) Roll pitch angles. The readings of the roll and pitch angle of the gyroscope reveals how fast the robot is capable of reorienting and recovering from a fall. (**c**) Snapshots of fall recovery on various terrains with different initial postures. (**c1**) Carpet. (**c2**) Grass. (**c3**) Mattress. (see Supplementary Video S2 for the corresponding video. Video URL: https://youtu.be/TCY2-pvkbjg).

#### Uneven terrain

The uneven terrain is automatically generated using the same approach utilized by Yang et al.^31^. The terrain consists of interconnected inclined surfaces, each surface covers a squared area with width of $l = 0.1\;{\text{m}}$. We measure the unevenness of the terrain as the maximum height *h* of the hill peak. The robot is able to adapt to the uneven terrain to a certain extent before failing to perform fall recovery when terrain unevenness reaches $h = 0.15\;{\text{m}}$ (Fig. 4b1). The robot is able to locomote when unevenness reaches $h = 0.15\;{\text{m}}$ (Fig. 4a1).

#### Low friction

The policies are tested under slippery ground conditions with different friction coefficients. The policies are able to perform fall recovery with relatively high success rates even when the friction coefficient reduces to 0 (Fig. 4b2). The locomotion performance significantly drops when the friction coefficient reduces to 0.2 and the robot fails to track the desired velocity (Fig. 4a2).

#### Actuation delay

Fall recovery behaviors can be performed with delays as high as 30 ms (Fig. 4b3). Locomotion behaviors are more easily affected by the delay, and the policy struggles to follow the user-commanded velocity of 4 m/s when delay exceeds 6 ms (Fig. 4a3).

#### Load

We emulate the effect of external load by changing the mass of the robot base in simulation. The robot is added with additional mass from range (0 kg, 15 kg) with an interval of 2.5 kg. With heavier load, the harder it is for the robot to locomote and track the target velocity command (Fig. 4a4).

**Fig. 6:**
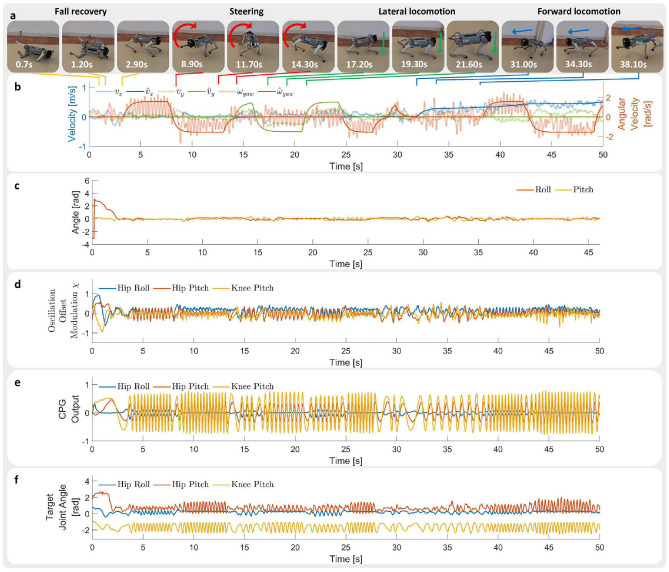
The robot is commanded to follow various velocity commands in real-world flat ground. Hip Roll represents the hip abduction/adduction joint, Hip Pitch represents the hip flexion/extension joint, Knee Pitch represents the knee flexion/extension joint. (**a**) Snapshots of the robot performing fall recovery, lateral locomotion, steering, and forward locomotion in real world. (**b**) Estimated velocity ($v_{x}$, $v_{y}$, $\omega _{yaw}$) and target velocity ($\hat{v}_{x}$, $\hat{v}_{y}$, $\hat{\omega }_{yaw}$). Note that the estimated velocity is calculated using Kalman filter and only serves as a reference. (**c**) Roll and pitch angles. The robot is able to perform fall recovery within 3.0s. (**d**) CPG oscillation offset $\chi$ of front left leg joints. (**e**) CPG output signal $\psi$ of front left leg joints. (**f**) Target joint angles of front left leg joints. (see Supplementary Video S2 for the corresponding video. Video URL: https://youtu.be/TCY2-pvkbjg).

#### Slope

We test the policies with downhill slopes of $-20^{\circ }$, $-15^{\circ }$, $-10^{\circ }$, and uphill slopes of $15^{\circ }$, $10^{\circ }$, $5^{\circ }$. The robot is able to adapt to larger slopes while walking downhill. The locomotion performance significantly drops on uphill slope of $15^{\circ }$, and downhill slope of $-20^{\circ }$ (Fig. 4a5).

It shall be noted that the training environment configuration was set up to be as simple as possible for the straightforwardness of replication. All policies were trained on flat terrain with a fixed friction coefficient of $\mu =1.0$.

The trained policies are able to traverse over uneven terrain, adapt to changes in ground friction, carry external load, and climb up slopes (See accompanying video). The policies exhibit highly dynamic motions while reacting to external uncertainties in the environment. Note that the policies are trained on flat ground and encountered no perturbations during the training phase.

**Fig. 7:**
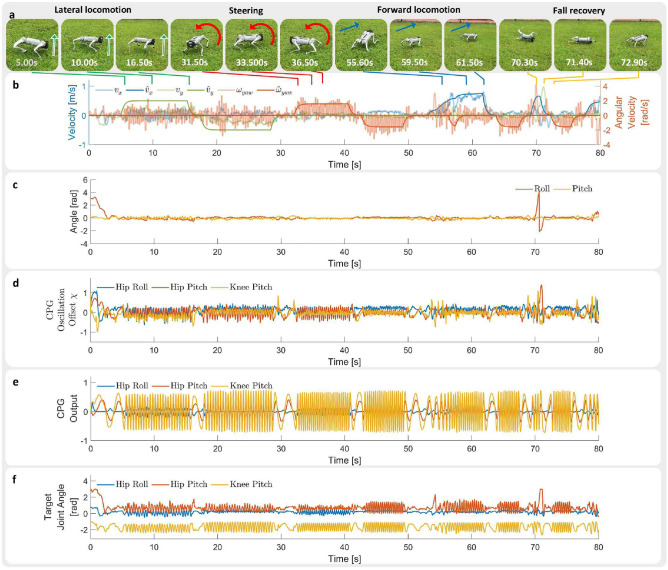
The robot is commanded to follow various velocity commands in real-world grasslands. (**a**) Snapshots of the robot performing fall recovery, lateral locomotion, steering, and forward locomotion in real world. (**b**) Measured velocity (${v}_{x}$, ${v}_{y}$, ${\omega }_{yaw}$) and target velocity ($\hat{v}_{x}$, $\hat{v}_{y}$, $\hat{\omega }_{yaw}$). Note that the measured velocity is estimated via Kalman filter. (**c**) Roll and pitch angles. (**d**) CPG oscillation offset $\chi$ of front left leg joints. (**e**) CPG output signal $\psi$ of front left leg joints. (**f**) Target joint angles of front left leg joints. (see Supplementary Video S2 for the corresponding video. Video URL: https://youtu.be/TCY2-pvkbjg).

### Hardware implementation

During hardware implementation, the velocity is estimated via a kalman filter and fused with the velocity estimated from foot movement. The policy loop is executed on an external laptop computer, while the PD loop is executed on the onboard computer of the robot. The laptop sends the position commands to the robot via wifi communication with an RTT of $2.657 \pm 0.566\;{\text{ms}}$.

We validate the policy’s fall recovery performance on (1) Grass, (2) Soft mattress, (3) Carpet (Fig. 5). Our policy can successfully perform fall recovery maneuvers from various initial postures, on different terrains (Fig. 5c1–c3). We applied more extreme external disturbances to induce fall failure and observed that the robot is able to perform an agile response to external disturbance and recover rapidly from falls into a stable upright posture (Fig. 5a and b).

We proceeded to command the robot to follow more diverse user target velocity commands on in the real world. We first start with the simple test of fall recovery and forward locomotion on flat terrain in the real world (Fig. 6). The robot starts from a supine posture with user given target velocity of 0. User target velocity is given after the robot recovers to standing posture. The robot is able to achieve a smooth transition from fall recovery to locomotion without discrete switches between different policies.

The robot also shows the capability to adapt and locomote on more challenging grasslands, demonstrating the generability and robustness of the learned policy (Fig. 7). The robot encountered fall failures during locomotion due to environmental uncertainties of the grassy terrain, but it is able to quickly recover and resume normal locomotion operation as can be seen in $70\;{\text{s}} - 73\;{\text{s}}$ time period in Fig. 7.

## Discussion

In this paper, we proposed a bio-inspired network architecture called MLP-CPG that utilizes the intrinsic rhythmicity of CPGs to generate rhythmic motor patterns for locomotion without relying on external phase input. We proposed a reformulation approach that transforms CPGs into stateless networks by exposing the internal states. With our approach, the parameters of CPGs can be optimized jointly with the MLP network using DRL without the need for a separate optimization algorithm.

The versatility of our MLP-CPG network is demonstrated by its multi-skill locomotion capability. We demonstrated our proposed network is capable of performing both rhythmic locomotion and non-rhythmic fall recovery within a single unified policy network and is able to freely transition between these two distinct motor behaviors. The multi-skill locomotion capability is validated through rigorous experiments in different real-world terrain scenarios on the Go1 robot hardware.

One of the core differences between the MLP-CPG framework and other control algorithms lies in how the control policies are structured and optimized. Previous works often rely on external phase inputs to generate rhythmic locomotion. These inputs are either manually designed or generated through additional components, both of which add complexity to the control system^1,2^ and introduce sub-optimality due to the manual design. In contrast, our approach generates the rhythmicity internally via the coherent RL training process, without relying on pre-programmed external signals, reducing system complexity and design effort.

Furthermore, many prior studies on CPG systems require separate design and training of the CPG and MLP components^17,18^. This separation between distinct modules increases the design effort, as well as the training and tuning time. In contrast, our framework enables the parameters of both the CPGs and MLP network to be optimized jointly via DRL. The simultaneous optimization of these components reduces the complexity in managing separate control modules and results in a more efficient training process, and more optimal and natural motions.

Additionally, previous approaches have often relied on dedicated policies for different tasks of locomotion and fall recovery, which required complex switching mechanisms to transition between behaviors^29,30^. In contrast, our approach integrates these distinct motor behaviors of rhythmic locomotion and non-rhythmic fall recovery into a single policy, making transitions between the two more natural and reducing manual effort in policy design.

In terms of performance, our framework can be compared with Ji et al.’s^29^ approach, which used the same Unitree Go1 robot hardware. Ji et al. achieved locomotion speeds of 1.5 m/s and demonstrated fall recovery on terrains such as sand, snow, grass, and tile. Our MLP-CPG framework achieved locomotion speeds of 1.0 m/s and performed fall recovery on soft mattress, grass, and carpet. While the locomotion speed in our study is slightly lower, our framework has both locomotion and fall recovery policies integrated within a unified policy, whereas Ji et al. required two separate policies. This integration allows for more seamless and natural transitions between various behaviors.

Due to the computational power of the onboard computer, the policy is implemented on an external computer, while sending commands to the onboard computer via wifi, leading to delays introduced by wireless communication. For future work, we plan to address this issue by upgrading the onboard computer and deploying the policy directly onboard the robot to eliminate the delay caused by wireless communication and improve performance.

The current policy relies solely on proprioception for blind locomotion and lacks visual input which hinders the policy’s capability to navigate over terrains that require precise footstep placements such as stairs and gaps with significant height variations. To address this limitation, future work will incorporate visual information into the framework to enable the policy to adjust the footstep placement for complex terrains.

Recent Studies have proposed training neural networks to provide more accurate estimations of error-prone information such as body velocity, foot contact and foot height^5,32^. Leveraging recent advancements in learning-based techniques for state estimations, future work will involve improving the policy’s performance by adopting a learning-based velocity estimator to replace the current Kalman filter-based velocity estimator.

## Methods

### Robot platform and simulation setup

The Unitree Go1 quadruped robot is used as the robot platform in simulation and real-world experiments. The Go1 robots weighs 12 kg with a dimension of around 645 $\times$ 280 $\times$ 400 mm while standing at its factory default posture. The joint motors have a max instataneous torque of 23.70 N m at the Hip Roll and Hip Pitch, and 35.55 N m at the Knee Pitch.

The policy is implemented on an external computer and sends commands to the Unitree Go1 onboard NVIDIA Jetson Nano computer via wifi. The external computer is a laptop with an i7-9750H CPU and a GTX 1660Ti Graphics card.

Pybullet is chosen as the physics engine for the simulation environment. The physics loop of the simulator runs at 500 Hz.

### CPG controller

The CPGs within this work are modeled based on the Kuramoto model^33^. Each oscillator corresponds to a joint motor.

1 $$\begin{aligned} \begin{aligned} \dot{\theta }_{i}^{t}&= 2 \pi f^{t}+\sum _{j}\varepsilon _{ij}sin(\theta _{j}^{t-1}-\theta _{i}^{t-1}-\phi _{ij})+\xi _{i}^{t},\\ \dot{r}_{i}^{t}&= \gamma \left( (\eta _{i}+\kappa _{i}^{t}) - (r_{i}^{t-1}) \right) ,\\ \psi _{i}^{t}&= r_{i}^{t} cos(\theta _{i}^{t}),\\ \mu _{i}^{t}&=\psi _{i}^{t} +\chi _{i}^{t}+o_{i},\\ p_{i}^{t}&= \frac{U_{i}-L_{i}}{1-(-1)}(\tanh (\mu _{i}^{t})-(-1))+L_{i}, \end{aligned} \end{aligned}$$

where $\theta _{i}^{t}$ and $r_{i}^{t}$ represent the phase and amplitude of the oscillator of the i-th motor at timestep *t*, $\dot{\theta }_{i}^{t}$ and $\dot{r}_{i}^{t}$ are their corresponding derivative, $\varepsilon _{ij}$ and $\phi _{ij}$ are the coupling weight and phase bias between i-th and j-th oscillator, $\eta _{i}$ is the default amplitude, $\eta _{i}+\kappa _{i}^{t}$ is the desired amplitude, $o_{i}$ is a constant offset of the oscillation setpoint, $\gamma$ is a constant that determines the rising time for *r*, $\psi _{i}^{t}$ represents the output signal of the CPG. Feedback components consists of $\kappa _{i}^{t}$, $\chi _{i}^{t}$, $\xi _{i}^{t}$, $f^{t}$, where $\kappa _{i}^{t}$ adjusts the amplitude, $\xi _{i}^{t}$ adjusts the phase, $\chi _{i}^{t}$ adjusts the oscillation setpoint, $f^{t}$ adjusts the frequency of the oscillator at timestep *t*. Finally, the CPG signal $\psi _{i}^{t}$ is added with $\chi _{i}^{t}$ and $o_{i}$ to generate the final output $\mu _{i}^{t}$. The value of $\mu _{i}^{t}$ is bounded within $[-1, 1]$ with a $\tanh$ activation function, and is rescaled into the corresponding joint range $[L_{i}, U_{i}]$ to obtain the desired joint angle $p_{i}^{t}$. The range for the Hip Roll, Hip Pitch, and Knee Pitch joints are $[-1.047rad, 1.047rad]$, $[-0.663rad, 2.966rad]$, and $[-2.721rad, -0.837rad]$, respectively.

**Fig. 8:**
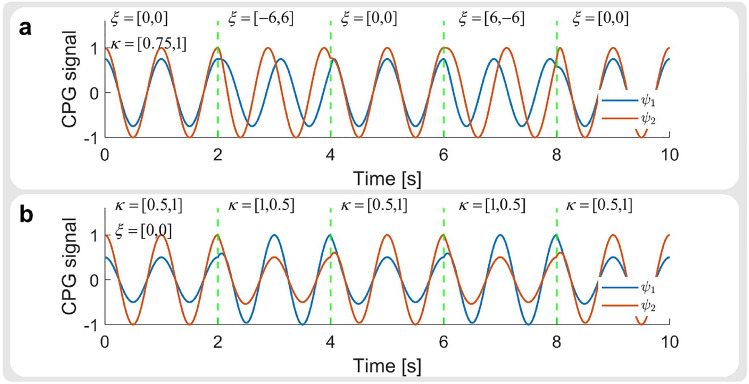
An example consisting of 2 coupled CPG oscillators. The values within $\phi _{ij}$, $\gamma$, and *dt* are set to 0, 5.0, and 0.01. The values of $\varepsilon _{12}$ and $\varepsilon _{21}$ are set to 6 while $\varepsilon _{11}$ and $\varepsilon _{22}$ are set to 0. The values within $\eta , \chi , o, \phi$ are set to 0. (**a**) and (**b**) illustrate how the phase and amplitude of the CPG can be adjusted by feedback components $\xi$ and $\kappa$.

The phase and amplitude $\theta _{i}^{t}$ and $r_{i}^{t}$ are obtained from their derivative values $\dot{\theta _{i}^{t}}$ and $\dot{r_{i}^{t}}$ using the following equation.

2 $$\begin{aligned} \begin{aligned} \theta ^{t}_{i}&= \theta ^{t-1}_{i}+\dot{\theta }^{t}_{i}dt,~ r^{t}_{i} = r^{t-1}_{i}+\dot{r}^{t}_{i}dt, \end{aligned} \end{aligned}$$

where *dt* is the duration of the timestep. An example of the CPG response can be seen in Fig. 8.

The robot joints are enumerated in the order of (1) Front left hip roll, (2) Front left hip pitch, (3) Front left knee pitch, (4) Front right hip roll, (5) Front right hip pitch, (6) Front right knee pitch, (7) Rear right hip roll, (8) Rear right hip pitch, (9) Rear right knee pitch, (10) Front Rear hip roll (11) Rear right hip pitch, (12) Rear right knee pitch.

Within the matrix of CPG parameters, only elements that correspond to the connection between adjacent joints are learned, while other elements are set to 0. The depiction of the connections between CPGs is shown in Fig. 10. The matrix of the coupling weight $\varvec{\varepsilon }$ and phase bias $\varvec{\phi }$ is shown in Eq. (3).

3 $$\begin{aligned} \begin{aligned} \varvec{\varepsilon }&=\small {\left\{ \begin{array}{lllllllllllll} 0 & \varepsilon _{1,2}& 0& \varepsilon _{1,4}& 0& 0& \varepsilon _{1,7}& 0& 0& \varepsilon _{1,10}& 0& 0 \\ \varepsilon _{2,1} & 0& \varepsilon _{2,3}& 0& 0& 0& 0& 0& 0& 0& 0& 0 \\ 0 & \varepsilon _{3,2}& 0& 0& 0& 0& 0& 0& 0& 0& 0& 0 \\ \varepsilon _{4,1} & 0& 0& 0& \varepsilon _{4,5}& 0& \varepsilon _{4,7}& 0& 0& \varepsilon _{4,10}& 0& 0 \\ 0 & 0& 0& \varepsilon _{5,4}& 0& \varepsilon _{5,6}& 0& 0& 0& 0& 0& 0 \\ 0 & 0& 0& 0& \varepsilon _{6,5}& 0& 0& 0& 0& 0& 0& 0 \\ \varepsilon _{7,1} & 0& 0& \varepsilon _{7,4}& 0& 0& 0& \varepsilon _{7,8}& 0& \varepsilon _{7,10}& 0& 0 \\ 0 & 0& 0& 0& 0& 0& \varepsilon _{8,7}& 0& \varepsilon _{8,9}& 0& 0& 0 \\ 0 & 0& 0& 0& 0& 0& 0& \varepsilon _{9,8}& 0& 0& 0& 0 \\ \varepsilon _{10,1} & 0& 0& \varepsilon _{10,4}& 0& 0& \varepsilon _{10,7}& 0& 0& 0& \varepsilon _{10,11}& 0 \\ 0 & 0& 0& 0& 0& 0& 0& 0& 0& \varepsilon _{11,10}& 0& \varepsilon _{11,12} \\ 0 & 0& 0& 0& 0& 0& 0& 0& 0& 0& \varepsilon _{12,11}& 0 \\ \end{array}\right\} }\\ \varvec{\phi }&=\small {\left\{ \begin{array}{lllllllllllll} 0 & \phi _{1,2}& 0& \phi _{1,4}& 0& 0& \phi _{1,7}& 0& 0& \phi _{1,10}& 0& 0 \\ \phi _{2,1} & 0& \phi _{2,3}& 0& 0& 0& 0& 0& 0& 0& 0& 0 \\ 0 & \phi _{3,2}& 0& 0& 0& 0& 0& 0& 0& 0& 0& 0 \\ \phi _{4,1} & 0& 0& 0& \phi _{4,5}& 0& \phi _{4,7}& 0& 0& \phi _{4,10}& 0& 0 \\ 0 & 0& 0& \phi _{5,4}& 0& \phi _{5,6}& 0& 0& 0& 0& 0& 0 \\ 0 & 0& 0& 0& \phi _{6,5}& 0& 0& 0& 0& 0& 0& 0 \\ \phi _{7,1} & 0& 0& \phi _{7,4}& 0& 0& 0& \phi _{7,8}& 0& \phi _{7,10}& 0& 0 \\ 0 & 0& 0& 0& 0& 0& \phi _{8,7}& 0& \phi _{8,9}& 0& 0& 0 \\ 0 & 0& 0& 0& 0& 0& 0& \phi _{9,8}& 0& 0& 0& 0 \\ \phi _{10,1} & 0& 0& \phi _{10,4}& 0& 0& \phi _{10,7}& 0& 0& 0& \phi _{10,11}& 0 \\ 0 & 0& 0& 0& 0& 0& 0& 0& 0& \phi _{11,10}& 0& \phi _{11,12} \\ 0 & 0& 0& 0& 0& 0& 0& 0& 0& 0& \phi _{12,11}& 0 \\ \end{array}\right\} }\\ \end{aligned} \end{aligned}$$

**Fig. 9:**
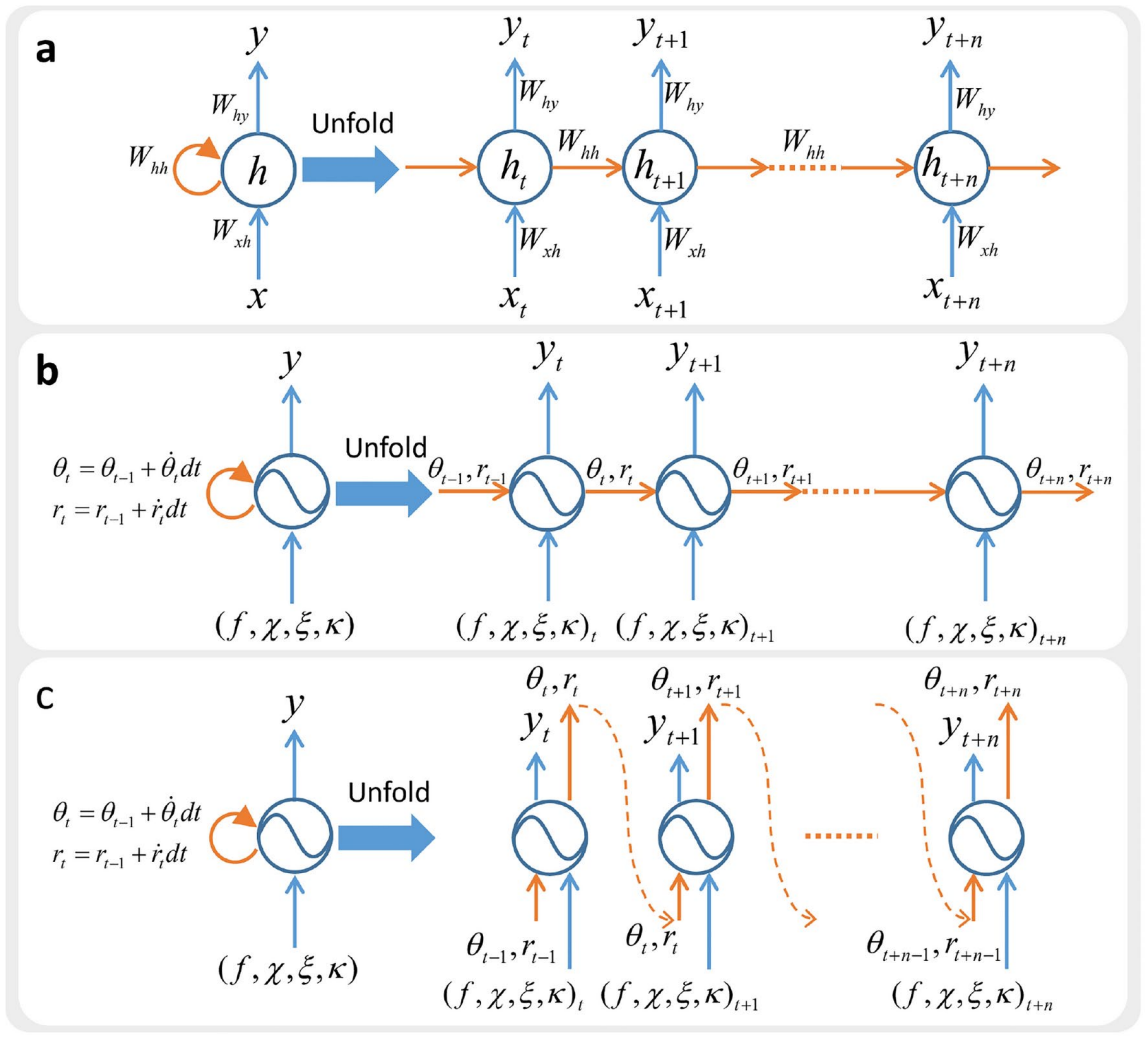
Illustration of RNN and CPGs. (**a**) Unfolded RNNs. (**b**) Unfolded CPGs. (**c**) Our reformulation approach transforms CPGs into stateless networks by exposing the hidden states as network inputs and outputs.

### Reformulating hidden states in CPGs

Within Recurrent Neural Networks (RNN), the hidden states of the previous timestep have to be passed to the current timestep and combined with the input of the current timestep to compute the current output (Fig. 9). The transition between the consecutive hidden states of an RNN neuron is governed by $W_{hh}$ (Fig. 9a). RNN requires BPTT to be trained. CPGs can be viewed as a type of RNN and are recurrent in nature, as they require hidden states to propagate information through time (Fig. 9b). The transition between consecutive hidden states of a CPG is determined by Eqs. (1) and (2).

We dealt with the recurrent nature of CPGs by proposing a reformulation approach that exposes the hidden states through network inputs and outputs. The previous hidden states will be passed as network inputs, and the current hidden states will be passed as network outputs. With our proposed reformulation approach, the CPG network is essentially transformed into a fully-differentiable stateless feedforward network (Fig. 9c).

In contrast to previous work where the parameters of the CPG controllers and MLP network are optimized with separate algorithms, our reformulated CPG network is differentiable and can be optimized jointly with MLP network within the same DRL paradigm without BPTT^25,26^.

**Fig. 10:**
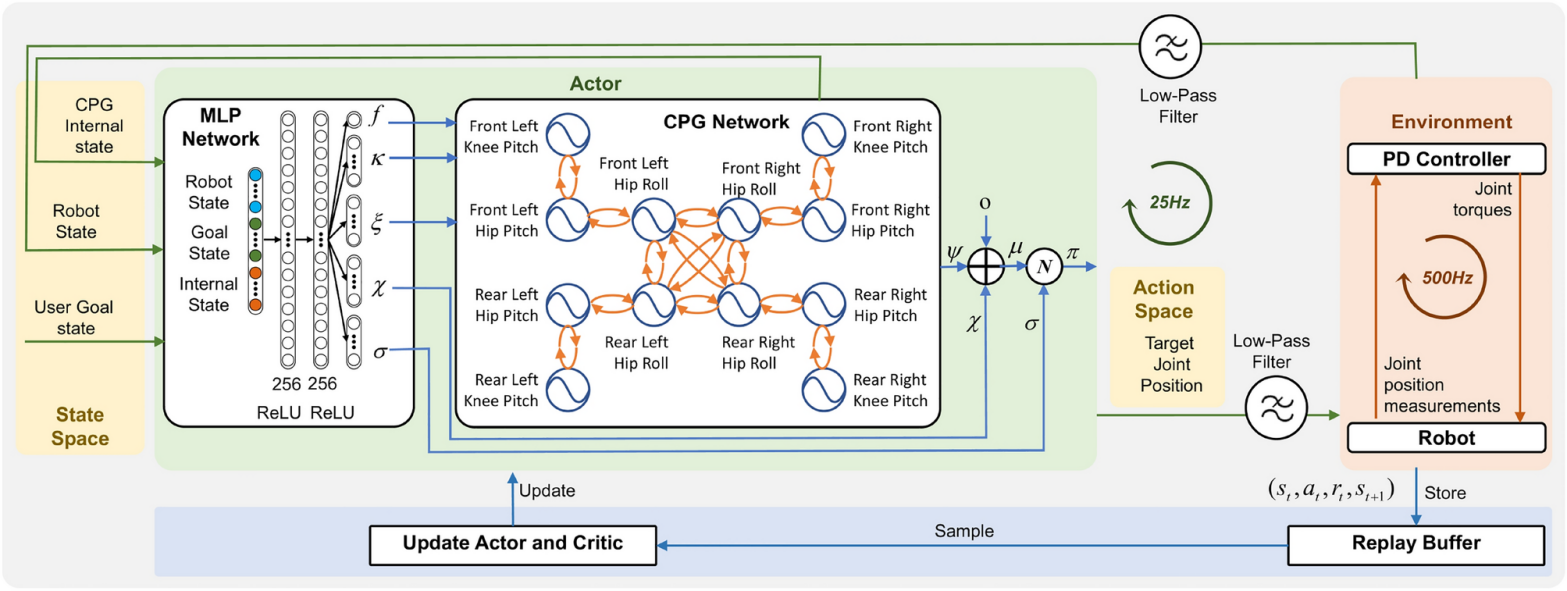
Control framework overview. The MLP-CPG network generates target joint positions at 25 Hz. The PD controller functions at 500 Hz and converts the joint angles to joint torques. The physics simulation runs at 500 Hz. The torque commands are held during the physics loop until updated.

Campanaro et al. have proposed a similar approach that dealt with the recurrent properties of CPGs by reformulating the CPG into a fully-differentiable stateless network, and thus allowing end-to-end training of CPGs alongside MLP. They demonstrated the proof-of-concept on a 2DoF leg constrained to move along the z-axis and learned successful hopping behaviors^24^. We extended upon the idea of reformulating CPG network into a stateless network and proposed our MLP-CPG framework. We have added a residual signal $\chi$ to adjust the oscillation setpoint to enable the generation of non-rhythmic movements. Furthermore, we have successfully implemented it on a 12-DOF quadruped robot in the real world.

### Network architecture

The MLP-CPG network consists of two separate modules, one rhythmic module responsible for generating rhythmic signals, and another non-linear feedback module responsible for processing sensory feedback (Fig. 10).

#### Rhythmic module

The rhythmic module contains 12 CPG neurons, each neuron represents a joint. As all four legs are identical, the value of the coupling parameters within $\varvec{\varepsilon }$ and $\varvec{\phi }$ that corresponds to the connection between hip pitch, and knee pitch joints are shared, i.e.

4 $$\begin{aligned} \begin{aligned} \phi _{2,3} = \phi _{5,6} = \phi _{8,9} = \phi _{11,12},\\ \phi _{3,2} = \phi _{6,5} = \phi _{9,8} = \phi _{12,11},\\ \varepsilon _{2,3} = \varepsilon _{5,6} = \varepsilon _{8,9} = \varepsilon _{11,12},\\ \varepsilon _{3,2} = \varepsilon _{6,5} = \varepsilon _{9,8} = \varepsilon _{12,11}.\\ \end{aligned} \end{aligned}$$

By having the same coupling parameters, the trajectory pattern of each foot will remain identical, encouraging the policy to learn a more consistent and symmetric gait pattern.

The values of the CPG parameters are shown in Table 2. While the coupling parameters are optimized with DRL, $\gamma$, *dt*, $\varvec{\eta }$, $\varvec{o}$ are manually set. Default amplitude $\varvec{\eta }$ is set with larger than 0 values to encourage the generation of rhythmic movements of the joint. The joint offset $\varvec{o}$ is a constant offset set to allow the robot to stand in a nominal upright posture when the values of $\varvec{\psi }$ and $\varvec{\chi }$ are 0 (Table 2).

#### Feedback module

The feedback module is an MLP network with 2 hidden layers of size 256 with $\tanh$ activation function. The MLP receives state inputs and regulates the CPG network via feedback components *f*, $\varvec{\kappa }$, $\varvec{\xi }$, $\varvec{\chi }$. The MLP also outputs the covariance $\varvec{\sigma }$ of the stochastic policy. The output of the MLP-CPG network $\varvec{\mu }$ will be used as the mean of the stochastic policy $\varvec{\Pi }$, where $\varvec{\Pi }=\mathcal {N}(\varvec{\mu }, \varvec{\sigma }).$

The policy output $\varvec{\Pi }$ is bounded within $(-1,1)$ using $\tanh$, and re-scaled to the corresponding joint limits to be used as the target joint angles $\varvec{a}$. The stochastic policy $\varvec{\Pi }$ is used during training for exploration. During actual implementation, the deterministic mean $\varvec{\mu }$ is used instead of the stochastic policy $\varvec{\Pi }$ to produce the target joint angles $\varvec{a}$.

**Table 2:** CPG parameters.

Parameter	Value
*dt*	0.04 [s]
$\gamma$	4
$\varvec{\varepsilon }$	Learned via DRL
$\varvec{\phi }$	Learned via DRL
$\varvec{o}$	$[\varvec{m}, \varvec{m}, \varvec{m}, \varvec{m}]$, $\varvec{m} = [0, 0.28, -0.1]$
$\varvec{\eta }$	$[\varvec{n}, \varvec{n}, \varvec{n}, \varvec{n}]$, $\varvec{n} = [0, 0.5, 0.5]$

**Table 3:** Training hyperparameters.

Hyperparameter	Value	Hyperparameter	Value
Gradient update steps	4	Learning rate	3e−4
Target network update	0.001	Weight decay	1e−6
Temporal regularization $\lambda _{T}$	1e−4	Discount factor	0.95
Spatial regularization $\lambda _{S}$	1e−3	Batch size	128
Frequency loss $\lambda _{f}$	1e−2	Diff loss $\lambda _{diff}$	1e−2

### Training setup

#### Soft actor critic

We selected Soft Actor Critic (SAC) as the DRL algorithm for the training of our policy. SAC learns a policy by maximizing the expected return and the entropy. The optimization objective can be expressed as follows:

5 $$\begin{aligned} J_{SAC} (\pi ) = \sum _{t=0}^{T} \mathbb {E}_ {(s_t,a_t) \sim \rho _{\pi }} [r(s_t, a_t) +\alpha H(\pi (\cdot |s_{t}))], \end{aligned}$$

where $\pi$ is the policy, $\rho _{\pi }$ is the sample distribution, *r* is the reward, $s_t$ and $a_t$ are the state and action at time step *t* within the sample distribution, $\alpha$ is the temperature parameter, and $H(\pi )$ is the entropy. The temperature parameter determines the stochasticity of the policy and is tuned automatically during training to balance exploration and exploitation. The hyperparameters can be seen in Table 3.

#### Q network update

Two Q networks $Q_{p_{1}}$ and $Q_{p_{2}}$ are trained using Double Q Learning to reduce the overestimation of the Q-value^34^. For each Q network $Q_{p_{1}}$ and $Q_{p_{2}}$, there is a separate target Q network $Q_{p'_{1}}$ and $Q_{p'_{2}}$ that slowly tracks the Q value function to improve learning stability^35^. The minimum of the two estimates from the target Q networks is used as the target update for Q-Learning:

6 $$\begin{aligned} y=r+\gamma (\min \limits _{i=1,2}Q_{p'_{i}}(s,a) ), \end{aligned}$$

where *r* is the reward, $\gamma$ is the discount factor, *s* is the state, *a* is the actor action, and *y* is the target update for the Q value.

The parameters for Q networks $Q_{p_{1}}$ and $Q_{p_{2}}$ are updated by minimizing the loss:

7 $$\begin{aligned} L_{Q_{p_{i}}}=\Sigma (y-Q_{p_{i}}(s,a)), \end{aligned}$$

and the parameters for target Q networks $Q_{p'_{1}}$ and $Q_{p'_{2}}$ are updated by:

8 $$\begin{aligned} p'_{i}=(1-\tau )p'_{i}+\tau p_{i}, \end{aligned}$$

where $p_{i}$ and $p'_{i}$ are the parameters of the original Q network and target Q network respectively, and $\tau$ is the target update coefficient that determines how fast the target network is updated to track the original Q value.

The Q networks contain 2 hidden layers of size 256 with $\tanh$ activation function and receive state and the actor action as inputs.

#### Generating smooth action

We introduce temporal and spatial regularization terms to encourage the learning of smoother actions^36^. We only regularize the oscillation setpoint feedback component $\chi$ of the network output.

9 $$\begin{aligned} \begin{aligned} L_{T} = \left\| \chi (s_{t})-\chi (s_{t+1}) \right\| ^{2}_{2}, L_{S} = \left\| \chi (s_{t})-\chi (\hat{s}_{t}) \right\| ^{2}_{2}\\ \end{aligned} \end{aligned}$$

The spatial regularization loss minimizes the difference between the action generated under observed state $s_{t}$ and perturbed state $\hat{s}_{t} \sim \mathcal {N}(s_{t}, \delta )$, where $\delta$ is the standard deviation. The temporal regularization loss minimizes the difference between the action under the current state $s_{t}$ and the next state $s_{t+1}$.

We also include a term to minimize the difference between the current measured joint angles and the target joint angles^1^,

10 $$\begin{aligned} \begin{aligned} L_{diff} = \left\| a(s_{t})-q_{m} \right\| ^{2}_{2}, \end{aligned} \end{aligned}$$

where $a(s_{t})$ is the target joint angles calculated from the deterministic mean $\mu$, and $q_m$ are the measured joint angles. As the framework uses PD control to generate joint torque, the loss $L_{diff}$ minimizes the applied joint torques by minimizing the difference between measured and target joint angles, thus generating smooth robot motions.

#### Step frequency

We design a reference function for the step frequency.

11 $$\begin{aligned} \begin{aligned} f_{ref}&= clip(max(4\left| \hat{v}_{x}\right| ,5\left| \hat{v}_{y}\right| ,2\left| \omega \right| ),0,5), \\ \end{aligned} \end{aligned}$$

where $f_{ref}$ is the step frequency, $\omega$ is the target turning rate, $\hat{v}_{x}$ and $\hat{v}_{y}$ are the target forward and lateral velocity, respectively. The maximum value of the three elements is selected and clipped within (0,5). The step frequency has to increase in response to locomotion velocity and turning velocity accordingly. We introduce loss function $L_{f}$ to encourage the MLP network to track the reference frequency $f_{ref}$.

**Table 4:** The basic definitions of the mathematical notation used in the equations for the reward terms.

Notation	Definition
$\varvec{\varphi }_{b}$	Projection of the gravity vector in the robot base frame
$h_{b}$	Robot base height (*z*) in the world frame
$v_{x}$	Forward velocity of the robot base along robot heading
$v_{y}$	Lateral velocity of the robot base along robot heading
$v_{z}$	Vertical linear velocity of the robot base
$\omega _{roll}$	Roll angular velocity of the robot base
$\omega _{pitch}$	Pitch angular velocity of the robot base
$\omega _{yaw}$	Yaw angular velocity of the robot base
$\varvec{\tau }$	Torque of joints
$\varvec{q}$	Angle of joints
$\varvec{\dot{q}}$	Velocity of joints
$(\hat{\cdot })$	Desired quantity of placeholder property $(\cdot )$
$\varvec{p}_{f, n}$	The n-th foot horizontal coordinates in the world frame
$\varvec{p}_{b}$	Base horizontal coordinates in the world frame
$\varvec{p}_{h, n}$	The n-th hip horizontal coordinates in the world frame
$h_{f, n}^{w}$	Height of n-th foot in world frame
$v_{f, n}^{w}$	Velocity of n-th foot in world frame

**Table 5:** Detailed description of task reward terms.

Reward term	Symbol
Forward velocity	$\frac{8}{31} \times K(v_{x}, \hat{v}_{x}, -4.6)$
Lateral velocity	$\frac{4}{31} \times K(v_{y}, \hat{v}_{y}, -4.6)$
Vertical velocity	$\frac{1}{31} \times K(v_{z}, 0, -4.6)$
Roll velocity	$\frac{1}{31} \times K(\omega _{roll}, 0, -1.87)$
Pitch velocity	$\frac{1}{31} \times K(\omega _{pitch}, 0, -1.87)$
Yaw velocity	$\frac{4}{31} \times K(\omega _{yaw}, \hat{\omega }_{yaw}, -1.87)$
Base orientation	$\frac{2}{31} \times K(\varvec{\varphi }_{b}, [0,0,-1], -2.35)$
Base height	$\frac{2}{31} \times K(h_{b}, \hat{h}_{b}, -51.17)$
Joint torque	$\frac{1}{31} \times K(\varvec{\tau }, 0, -0.001)$
Joint velocity	$\frac{1}{31} \times K(\varvec{\dot{q}}, 0, -0.026)$
Ground contact	$$\frac{1}{{31}} \times \left\{ {\begin{array}{*{20}l} {0,\quad \quad \quad {\text{upper}}\;{\text{body}}\;{\text{contact}}\;{\text{with}}\;{\text{ground}}} \hfill \\ {1,} \hfill \\ \end{array} } \right.$$
Self-collision	$$\frac{1}{{31}} \times \left\{ {\begin{array}{*{20}l} {0,\quad \quad {\text{self}}\;{\text{collision}}} \hfill \\ {1,} \hfill \\ \end{array} } \right.$$
Swing & stance	$\frac{2}{31} \times \frac{1}{4} \sum _{4}^{n=1}K((h_{f, n}^{w}-\hat{h}_{f, n}^{w})v_{f, n}^{w}, 0, -460)$
Body placement	$\frac{1}{31} \times K(\frac{1}{4} \sum _{4}^{n=1}(\varvec{p}_{f, n}^{w}), \varvec{p}_{b}^{w}, -51.17)$
Foot placement	$\frac{1}{31} \times \frac{1}{4} \sum _{4}^{n=1}K(\varvec{p}_{f, n}, \varvec{p}_{h, n}, -51.17)$

12 $$\begin{aligned} \begin{aligned} L_{f}&= \left\| f(s_{t})-f_{ref} \right\| ^{2}_{2}\\ \end{aligned} \end{aligned}$$

The frequency loss $L_{f}$, the temporal regularization loss $L_{T}$, and spatial regularization loss $L_{S}$ are added to the loss function of the SAC $L_{SAC} = -J_{SAC}$.

13 $$\begin{aligned} L = & L_{{SAC}} + \lambda _{T} L_{T} + \lambda _{S} L_{S} + \lambda _{{diff}} L_{{diff}} + \lambda _{f} L_{f} \\ \lambda _{T} = & 0.0001,\lambda _{S} = 0.001,\lambda _{{diff}} = 0.01,\lambda _{f} = 0.01 \\ \end{aligned}$$

### Reward design

Radial basis function (RBF) kernels are used in the reward design. The output range of RBF is bounded within (0, 1). The formulation of the RBF kernel is shown as follows:

14 $$\begin{aligned} K(x, \widehat{x}, c)=\exp \left( c(\hat{x}-x)^{2}\right) , \end{aligned}$$

where *x* is the physical quantity for the evaluation, $\hat{x}$ is the desired value, and *c* is the parameter that controls the width of the RBF.

**Fig. 11:**
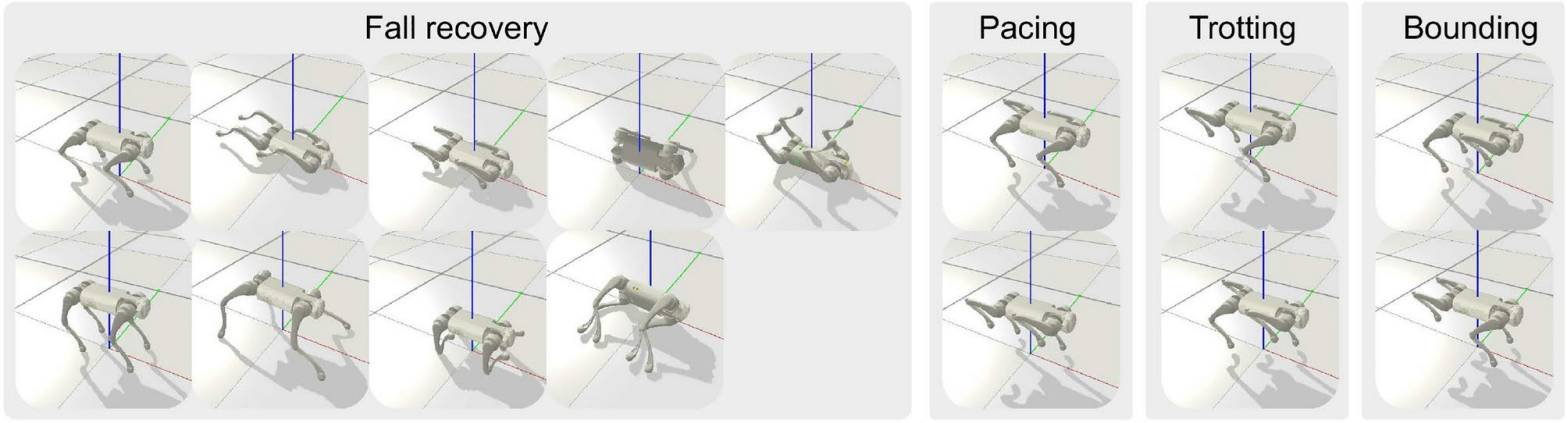
Robot initialization configurations.

The reward is the weighted sum of individual reward terms, each governing a different physical aspect (Tables 4 and 5). The reward terms consist of (1) Forward velocity tracking; (2) Lateral velocity tracking; (3) Vertical velocity penalty; (4) Yaw velocity tracking; (5) Roll velocity penalty; (6) Pitch velocity penalty; (7) Base orientation; (8) Base height; (9) Body placement; (10) Foot placement; (11) Swing and Stance foot; (12) Joint torque regularization; (13) Joint velocity regularization; (14) Body-ground contact penalty; (15) Self-collision penalty. The Swing and stance foot reward penalizes the movement of the stance foot close to the ground while encouraging the movement of the swing foot far from the ground. The body placement reward encourages the Center of Mass (COM) of the robot to be placed close to the center of the support polygon. The foot placement reward encourages the feet to be placed close beneath the hip joint.

### State space and action space

The state space consists of the robot proprioceptive states $S_{R}\in \mathbb {R}^{36}$, the internal states of the CPGs $S_{I}\in \mathbb {R}^{24}$, and the goal states provided by the user $S_{G}\in \mathbb {R}^{3}$.

The robot proprioceptive states consist of base linear velocity in robot heading frame $\varvec{v}_{xyz}\in \mathbb {R}^{3}$, base angular velocity $\varvec{\omega }_{rpy}\in \mathbb {R}^{3}$, orientation vector $\varvec{\varphi }_{b}\in \mathbb {R}^{3}$, joint position $\varvec{q}\in \mathbb {R}^{12}$, and joint velocity $\varvec{\dot{q}}\in \mathbb {R}^{12}.$ The goal states consist of the target forward velocity $\hat{v}_{x}\in \mathbb {R}^{1}$, target lateral velocity $\hat{v}_{y}\in \mathbb {R}^{1}$, and the target yaw turning rate $\hat{\omega }_{z}\in \mathbb {R}^{1}$. The internal states consist of the amplitude $\varvec{r}\in \mathbb {R}^{12}$ and phase $\varvec{\theta }\in \mathbb {R}^{12}$ of the 12 CPG oscillators. A mod operation is used to bound the phase within the range $[0, 2\pi ]$. The observation states $S_{R}\in \mathbb {R}^{36}$ are filtered by a low-pass Butterworth filter with a cut-off frequency of 10 Hz.

The action space $a_{t}$ contains the target joint angles $\hat{q}$. A low-pass Butterworth filter with a cut-off frequency of 8 Hz is applied to the target joint angles to encourage smooth actions. PD controllers are used to calculate joint torques $\tau$ from the target joint angles $\hat{q}$, measured joint angles *q*, and measured joint velocities $\dot{q}$ using the following equation $\tau = K_{p}(\hat{q}-q)+K_{d}(0-\dot{q})$, where $K_{p} = 40$, $k_{d} = 2$. The policy runs at 25 Hz while the PD loop runs at 500 Hz.

### Exploration setup

#### Initialization

The initial forward velocity, lateral velocity, and yaw angular velocity are sampled from $U(-5,5),$ $U(-1,1)$, and $U(-\pi , \pi )$ respectively. We adopted nine robot initialization configurations for fall recovery presented in previous work^31^. We designed an additional six robot configurations of pacing, trotting, and bounding for locomotion (Fig. 11).

The CPG amplitude and phase are initialized from $U(0,\frac{\pi }{4})$ and $U(0,2\pi )$, respectively. The target forward velocity, target lateral velocity, and target yaw turning rate are initialized from $U(-1,5)$, $U(-1,1)$, and $U(-\pi , \pi )$, respectively.

#### Early termination

It is normal for the robot to fall during training and reach a fail state that is difficult to recover from. During training, samples of the robot struggling to get up will fill up the replay buffer. Such samples are uninteresting to explore as they reduce the sample diversity and contribute little to learning. We set a time limit of 10*s* to terminate the episode early and reset the robot state.

## Supplementary Information

Supplementary Information 1.

Supplementary Information 2.

## Data Availability

All data used and analyzed within this paper are available from the corresponding author upon reasonable request.

## Code availability

The code is publicly available in the Zenodo repository^37^. The doi of the code is https://doi.org/10.5281/zenodo.14991755. The license is GNU General Public License v.3.0.
